# Low-Cost DLW Setup for Fabrication of Photonics-Integrated Circuits

**DOI:** 10.3390/mi17010125

**Published:** 2026-01-19

**Authors:** André Moreira, Alessandro Fantoni, Miguel Fernandes, Jorge Fidalgo

**Affiliations:** 1 Departamento de Engenharia Electrónica e Telecomunicações e de Computadores, Instituto Superior de Engenharia de Lisboa (ISEL), 1959-007 Lisboa, Portugal; a47125@alunos.isel.pt (A.M.); mfernandes@deetc.isel.ipl.pt (M.F.); jfidalgo@deect.isel.ipl.pt (J.F.); 2 Center of Technology and Systems (UNINOVA-CTS) and Associated Laboratory of Intelligent Systems (LASI), Quinta da Torre, Monte da Caparica, 2829-516 Caparica, Portugal

**Keywords:** photonic integrated circuit, DWL, lithography, laser, piezo stages, SU-8, polymer structures, photonics, polymer based, optical fabrication, prototyping, photoresist, integrated optics, microfabrication, micromachines

## Abstract

The development of photonic-integrated circuits (PICs) for data communication, sensing, and quantum computing is hindered by the high complexity and cost of traditional fabrication methods, which rely on expensive equipment, limiting accessibility for research and prototyping. This study introduces a Direct Laser Writing (DLW) system designed as a low-cost alternative, utilizing an XY platform for precise substrate movement and an optical system comprising a collimator and lens to focus the laser beam. Operating on a single layer, the system employs SU-8 photoresist to fabricate polymer-based structures on substrates such as ITO-covered glass. Preparation involves thorough cleaning, spin coating with photoresist, and pre- and post-baking to ensure material stability. This approach reduces dependence on costly infrastructure, making it suitable for academic settings and enabling rapid prototyping. A user interface and custom slicer process standard .dxf files into executable commands, enhancing operational flexibility. Experimental results demonstrate a resolution of 10 µm, with successful patterning of structures, including diffraction grids, waveguides, and multimode interference devices. This system aims to transform PIC prototype fabrication into a cost-effective, accessible process.

## 1. Introduction

Photonic Integrated Circuits (PICs) are foundational to advancements in high-speed optical communications [1], quantum computing [2], and biomedical sensing [3], offering significant advantages over conventional electronic systems. They are built from basic photonic structures such as waveguides, ring resonators, and optical couplers, which guide and manipulate photons to perform various functions. PICs enable practical applications like multiplexers with ultra-high bandwidth and minimal signal loss in telecommunications [4], and ultrasensitive lab-on-chip biosensors in healthcare [5].

Conventional photolithography for PICs fabrication requires the fabrication of a new set of masks for each iteration of device development creating long delays making it financially unfeasible for most universities [6]. As a result, students and researchers can design sophisticated photonic devices computationally, but these designs often remain confined to simulations with limited opportunities for experimental validation. Furthermore, emerging fields such as lab-on-a-chip diagnostics and quantum photonics demand rapid and iterative prototyping, a need incompatible with industrial-scale fabrication’s rigid workflows.

Direct Laser Writing (DLW) emerges as a promising alternative, combining two key advantages: low cost and maskless operation [7]. These features make DLW particularly suitable for academic environments where accessibility and affordability are essential. The aim of this work is to develop a lithography system for photonic circuit fabrication that can be implemented in academic contexts, considering parameters such as cost, simplicity, and usability. The system must remain compact, safe, and flexible while maintaining the minimum resolution required for the fabrication of polymer-based PICs.

The performance and scalability of PICs strongly depend on the choice of materials. Commonly used materials include silicon [8], polymers [9], lithium niobate [10], and III–V semiconductors such as Indium Phosphide (InP) and GaAs [11,12]. Silicon is the dominant platform due to its compatibility with CMOS technology [13] and low optical losses, whereas polymers offer flexibility and low-cost prototyping capabilities. Lithium niobate provides excellent electro-optic properties, and InP enables integration of both active and passive components, though at higher cost and complexity [14].

PICs enable a broad range of applications. In medicine, they are used as diagnostic tools due to their small size, high sensitivity, and rapid detection capabilities [15], with lab-on-a-chip platforms detecting molecular interactions in a label-free manner [16]. In telecommunications, PICs are employed in optical phased arrays with nanoantennas and phase shifters for signal steering and multiplexing [17]. In computing, PIC-based logic gates offer ultrafast operation and energy efficiency, reaching terahertz processing speeds [18].

The fabrication of PICs typically relies on lithography techniques such as photolithography, Electron Beam Lithography (EBL), and Direct Laser Writing (DLW) [19]. Photolithography is the most established and scalable method, transferring patterns through photomasks onto photoresist-coated substrates with high throughput and precision [20]. However, the requirement for cleanrooms, masks, and expensive alignment systems makes it inaccessible for low-budget laboratories. EBL, on the other hand, achieves nanometer-scale resolution by using a focused electron beam to directly write on electron-sensitive resists [21,22]. Despite its precision, EBL suffers from slow processing times and high equipment costs, making it unsuitable for routine prototyping.

DLW overcomes many of these limitations by directly writing the desired pattern into the photoresist using a focused laser beam, eliminating the need for photomasks [23]. The technique provides sufficient resolution for micrometer-scale PIC structures and allows researchers to fabricate custom designs with high flexibility and minimal infrastructure. By optimizing parameters such as laser wavelength, exposure time, and stage precision, DLW enables accessible, rapid, and low-cost prototyping of photonic devices [24,25].

Therefore, this research aims to address the limitations of traditional lithography by proposing a low-cost and accessible DLW-based system capable of fabricating polymer-based photonic circuits. The system targets academic environments where affordability, simplicity, and usability are crucial, providing a pathway to bridge the gap between simulation and experimental validation in photonic research.

Direct laser writing (DLW) has historically been limited to institutions with access to expensive femtosecond laser systems. State-of-the-art commercial DLW solutions, as demonstrated in academic studies, allow an impressive resolution of 300 nm [26].

However, recent literature describes several cost-effective approaches that dramatically lower the barrier to entry while maintaining useful resolution and functional capabilities. These technological advances are particularly important, as they allow iterative and rapid prototyping of complex devices, offering freedom of layout design and cost-effective integration of micro-optical and micro-electrical elements.

The most immediately affordable DLW implementations make use of low one-photon absorption (LOPA) in standard photoresists using visible-wavelength continuous-wave lasers. Reynoso-de la Cruz et al. [27] demonstrated a complete DLW system constructed for approximately USD $1000 using a green laser pointer at 532 nm with 50 mW output, a simple 5× microscope objective (NA 0.13), and a computer-controlled XYZ translation stage. The system employed SU8-2050 photoresist and achieved about 40 μm axial resolution.

A parallel implementation by Camarena-Chávez et al. [28] similarly used a commodity 532 nm laser pointer (65 mW) with 5× and 10× Motic achromatic objectives to pattern SU8-2050 photoresist, achieving optical resolution of 120 μm and mechanical positioning accuracy of 10 μm.

A commercial system relevant for comparison is the UV Direct Writer (UVW03) from Sejong Scientific Instruments [29], a still cost-effective maskless lithography platform using a 405 nm laser. This system focuses on linear waveguide fabrication, relying on pre-defined vector paths that allow only straight segments. While this approach can produce basic waveguide patterns, it is inherently limited when more flexible routing is required, as it cannot generate curved waveguides or non-linear layouts. In terms of resolution, the UVW03 achieves usable structures down to approximately 10 μm, and its maximum writing speed is around 600 μm/s, optimized for straight-path patterning.

When compared with the above-mentioned alternatives, the system presented in this work emphasizes flexibility in path generation and exposure control. Although the system writes at a slightly slower speed of 300 μm/s, this trade-off allows curved waveguides, which are essential for advanced photonic designs. The software-driven approach enables dynamic control over laser exposure and stage movement, ensuring that both straight and curved waveguides are effectively produced. As a result, the system can reliably fabricate waveguides with feature sizes down to 5 μm, delivering a versatile lithography platform for academic research and development. Its combination of curved-path capability, higher practical resolution, and software-controlled precision makes it particularly advantageous for applications that require complex waveguide routing and high-quality photonic structures.

## 2. Methods

**Figure 1:**
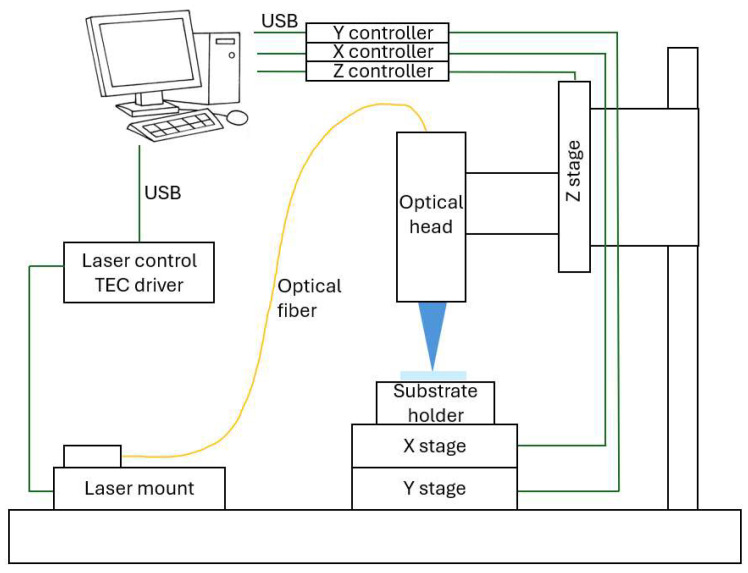
Proposed system overview.

The proposed Direct Laser Writing (DLW) system integrates optical, mechanical, and electronic components to fabricate polymer-based Photonic Integrated Circuits (PICs) in academic environments. A 405 nm laser (Thorlabs LP405-SF10, Thorlabs, Inc., Newton, NJ, USA) mounted in an LDM9LP holder (Thorlabs, Inc., Newton, NJ, USA), is coupled via monomode fiber to a TC25FC-405 collimator (Thorlabs, Inc., Newton, NJ, USA) and a C775TMD-405 aspheric lens (Thorlabs, Inc., Newton, NJ, USA), both secured in a KM100T tilt mount (Thorlabs, Inc., Newton, NJ, USA) and a Thorlabs SM1L20C lens tube (Thorlabs, Inc., Newton, NJ, USA) to ensure optical alignment. The optical head, including the lens assembly and the Z-axis Xeryon XLS-3-40-78 motor (Xeryon, Leuven, Belgium) is anchored to a vibration-damped Thorlabs DP12A post (Thorlabs, Inc., Newton, NJ, USA) via post clamps and custom aluminum supports, providing stability and focusing capability. The substrate is positioned on an XY platform driven by two XLS-1-40-78 motors (Xeryon, Leuven, Belgium) which trace geometric patterns under the stationary laser and optics assembly. Laser current and temperature are set via an IP250-BV analog controller (Thorlabs, Newton, NJ, USA) that is controlled by a custom built digital control board, and an MTD415T temperature controller (Thorlabs, Newton, NJ, USA) all interfaced with a software GUI (version 3.4). that converts design files into executable commands. The system architecture is summarized in Figure 1, illustrating the integration of optics, mechanics, electronics, and software.

The system integrates optomechanical hardware, and software components. An XY platform moves the substrate under the laser, tracing geometric patterns from design files, while a Z-axis maintains focus. Laser modulation controls exposure dose, and software converts designs into motor commands.

**Figure 2:**
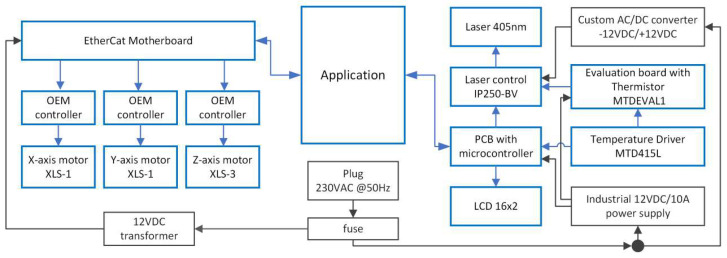
Block diagram of the proposed system.

The block diagram in Figure 2 summarizes the system architecture, highlighting active modules in blue and supporting/power elements in black. The software consists of a slicer, GUI, processing module, and initialization module. The slicer converts DXF files into executable commands, the GUI allows parameter adjustment and monitoring, and the processing module executes patterning by sending synchronized instructions to the motors and laser.

### 2.1. Mechanics

The mechanical system developed for the DLW setup is stable, precise, affordable, and easy to replicate, supporting the fabrication of polymer-based PIC in academic environments. Thorlabs mechanical components, such as posts, bases, and mounts, are used to support and secure the optical elements, ensuring mechanical stability and precise alignment using standard components. Xeryon piezoelectric motors were employed for the motion platform due to their resolution and cost. To complete the system, custom mechanical pieces were developed, including connectors to link the optics to the motors, an inclination adjustment mechanism, and a substrate holder. These custom components are made from aluminum due to its affordability and lightweight nature, which aligns with the system’s cost and load constraints, and were designed in SolidWorks (version 2025) and fabricated using CNC machining.

**Figure 3:**
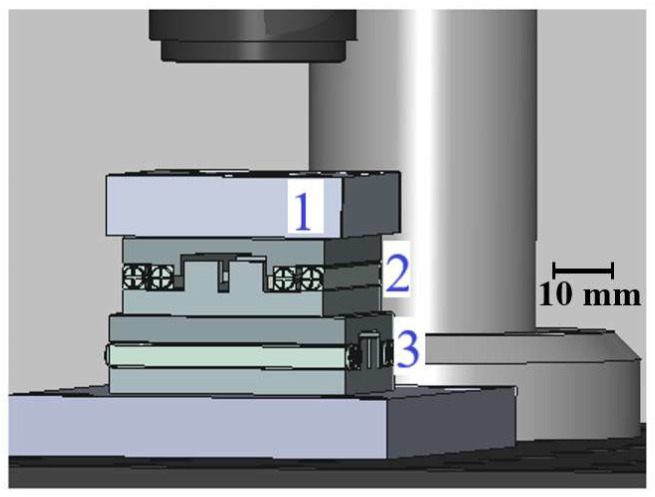
Substrate holder with X and Y motors. The substrate holder (1) is supported by two motors: X motor (2) and Y motor (3).

The substrate holder is constructed from black-painted aluminum to minimize optical reflections, featuring a V-shaped clamp and a corner alignment guide to ensure repeatable substrate positioning, with a weight of 41 g. The holder is supported by two Xeryon XLS-1-40-78 (Xeryon AG, Zürich, Switzerland) motorized translation stages, with the lower motor (Y) carrying the combined weight of the holder and the upper motor, totaling a weight of 92 g (Figure 3). The combined weight of the substrate holder and upper motor is well within the Y motor’s maximum rated capacity of 500 g, and its resolution is sufficient for the fabrication of polymer-based PICs. While this configuration is adequate for current applications, potential improvements include fine-tuning the motor parameters—such as acceleration, proportional gains, and mass compensation—to minimize vibrations and reduce settling time during positioning.

**Figure 4:**
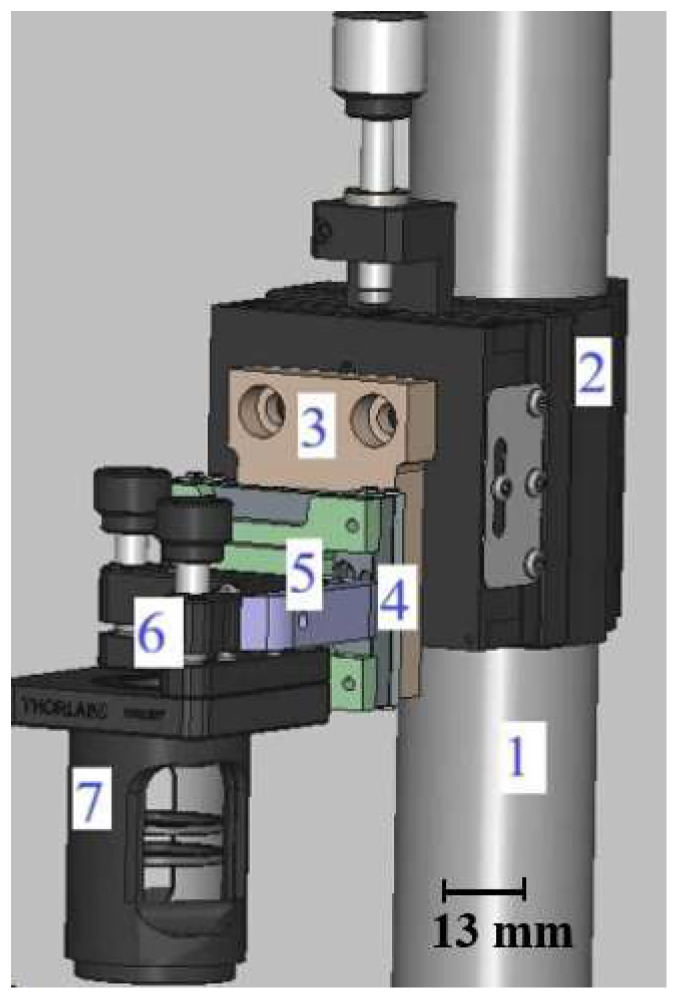
Optical head mechanical system with labeled components: (1) anti-vibration post, (2) post clamp, (3) custom aluminum link, (4) Z-axis motor, (5) support to optics assembly, (6) adjustable mount, (7) lens tube with collimator and focusing lens.

The optical head contains the collimator, laser, lens, and associated optics, and is attached to a Z-axis motor to adjust the position of the focal point relative to the substrate (Figure 4). This configuration enables vertical adjustment to achieve the minimum spot size.

The structure is anchored by an anti-vibration post (1), secured with a post clamp (2). A custom aluminum piece (3) links the clamp to the Z-axis motor (4), which moves the optical head. A second custom aluminum support (5) connects the motor to the laser optics assembly, which attaches to an adjustable mount (6) holding the lens tube (7) with collimator and focusing lens. The mount allows tip-tilt adjustments of the optical head in both the X and Y planes. Since the substrate is flat, these adjustments are used to control the symmetry of the laser beam, ensuring it forms a circular Gaussian spot on the substrate surface.

**Figure 5:**
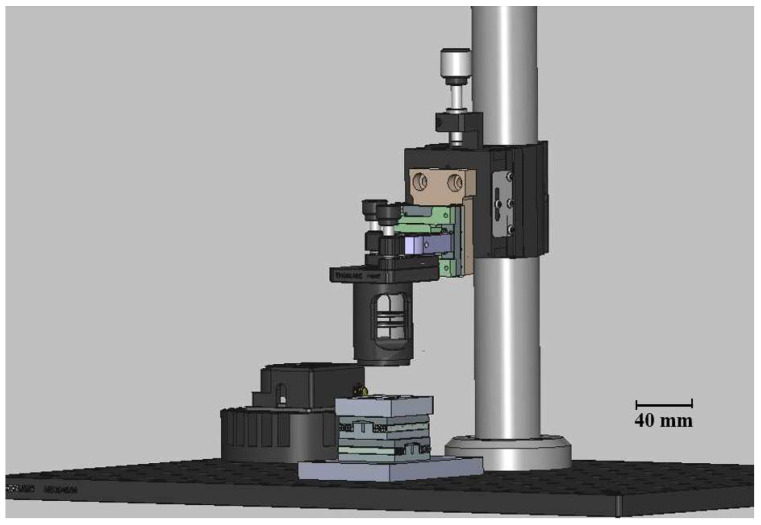
3D representation of the complete mechanical system developed for the DLW setup.

Figure 5 presents the full assembly of the mechanical system, including the motion stages, substrate holder, and optical head.

### 2.2. Optics

The optical system sensitizes the SU-8 photoresist using a 405 nm laser source. Although shorter wavelengths such as 355 nm can theoretically achieve smaller focused spot sizes, the 405 nm diode laser was selected due to its affordability and availability, aligning with the objective of developing a cost-effective DLW platform.

The optical setup consists of a laser module with an integrated single-mode fiber, a TC25FC-405 collimator (Thorlabs, Inc., Newton, NJ, USA) for transforming the diverging fiber output into a parallel beam, and an aspheric focusing lens (C775TMD-405, Thorlabs, Inc., Newton, NJ, USA) that concentrates the beam onto the substrate. The collimator allows mechanical adjustment to fine-tune beam divergence, while the focusing lens provides a 2.0 mm focal length and high numerical aperture, enabling the formation of a tightly focused spot suitable for high-resolution exposure. Both optical elements are mounted inside a threaded mechanical structure to ensure stability and repeatable alignment.

**Figure 6:**
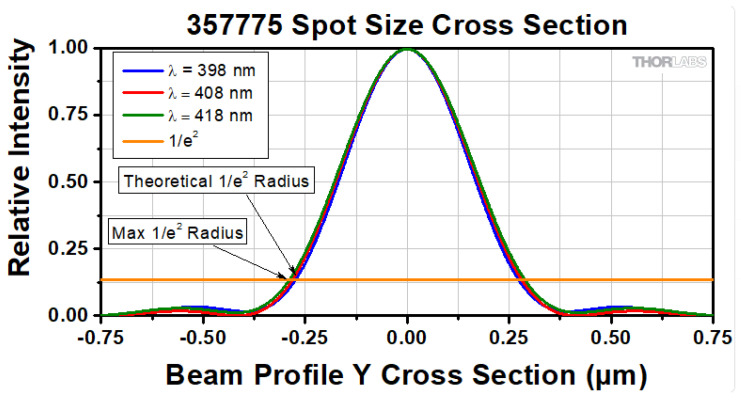
Aspheric lens C775TMD-405 focal point.

Figure 6 shows the spot cross section of the Thorlabs C775TMD-405 aspheric lens. At the focal plane, the beam forms a diffraction-limited spot with a waist of approximately 512 nm, determined by the lens aperture and focal length.

**Figure 7:**
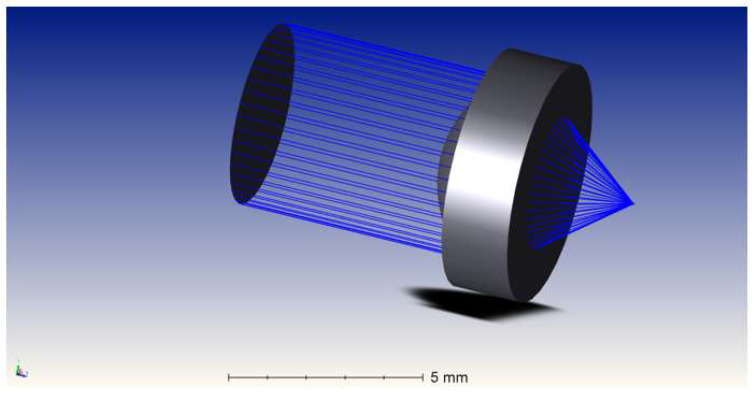
Simulation of laser propagation from the collimator through the aspheric lens to the focal point.

Beam propagation was simulated in Zemax OpticStudio (version 22.2, Zemax LLC, Kirkland, WA, USA) as reported in Figure 7 to evaluate the focusing behavior of the system. The simulation included the collimated Gaussian beam and the aspheric lens geometry. Results indicate that the optical system forms a near diffraction-limited spot, with an estimated waist of approximately 512 nm located at the focal plane.

**Figure 8:**
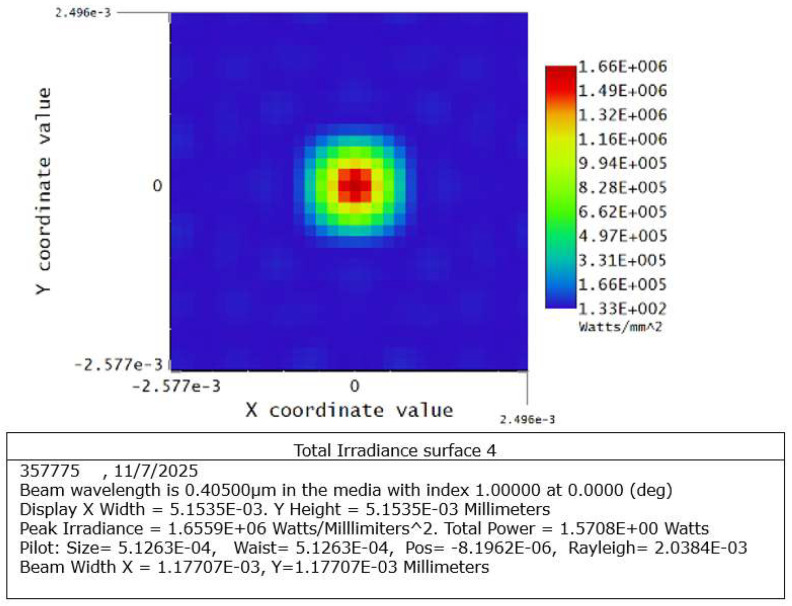
Simulated irradiance distribution at the focal point.

The simulation results also revealed a strong dependence on axial positioning (Figure 8). A defocus of approximately 10 µm increases the focused beam diameter by nearly a factor of five relative to the minimum waist, demonstrating the high sensitivity of the system to focal distance. This sensitivity presents a challenge during alignment, especially in the absence of an active feedback mechanism.

To mitigate misalignment effects, the optical head—including the collimator and focusing lens—is mounted on an XY-tilt platform. During setup, the beam is slightly defocused to make its profile visible, and tilt adjustments are manually carried out until a symmetric circular spot is achieved on the substrate. Once the alignment is correct, the system is returned to the focal position to obtain the minimum spot size.

### 2.3. Laser Control

Dedicated hardware has been implemented to control the LP405-SF10 laser diode (Thorlabs, Inc., Newton, NJ, USA), providing temperature regulation as well as analog and digital current control. Thermal management is ensured by the MTD415TE temperature driver (Thorlabs, Inc., Newton, NJ, USA) mounted on its evaluation board, which connects to the LDM9LP laser mount (Thorlabs, Inc., Newton, NJ, USA) via DB9 and interfaces with the IP250-BV analog current driver (Thorlabs, Inc., Newton, NJ, USA). The STATUS pin enables an interlock mechanism that shuts down the laser in case of over-temperature, protecting the device. Temperature sensing uses the mount’s 10kΩ NTC thermistor and TEC for active regulation, with PID parameters tuned via UART. The temperature is monitored on a custom PCB with the analog front-end and a microcontroller STM32F103 (STMicroelectronics, Geneva, Switzerland).

**Figure 9:**
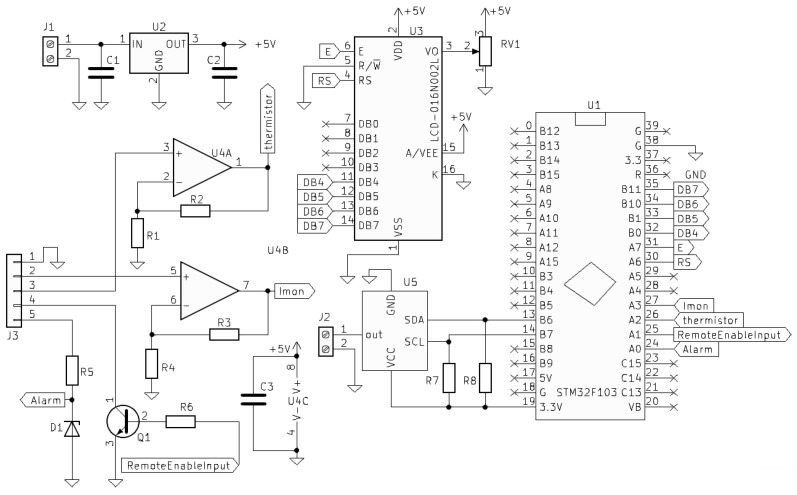
Schematic of the custom printed circuit board for digital laser control.

The LP405-SF10 diode is connected to the Thorlabs IP250-BV laser diode controller (Thorlabs, Inc., Newton, NJ, USA) using a 3-pin configuration (anode, cathode, and ground), with the integrated photodiode providing feedback for monitoring laser power. The IP250-BV is a 250 mA analog driver designed for blue laser diodes, offering Automatic Current Control (ACC) and Automatic Power Control (APC) modes. In ACC mode, it maintains a constant injection current, adjustable from 0 to 250 mA, while APC mode regulates laser power via photodiode feedback. The board allows analog modulation via a BNC connector (Amphenol RF, Wallingford, CT, USA) (0–10 VDC, 25 mA per 1 V) and can be enabled either by shorting pins J4-1 and J4-2 or by a two-state push-button on the board. Power is supplied via ±12 V DC power supply, and analog monitoring outputs provide real-time current and voltage indications. Digital control is implemented through a custom PCB using an MCP4725 DAC (Microchip Technology Inc., Chandler, AZ, USA) and NPN transistor to activate the laser, interfacing with an STM32F103 microcontroller (STMicroelectronics, Geneva, Switzerland). Current and temperature signals are amplified to match the ADC input range (3.3 V), and USB communication enables software control, as illustrated in Figure 9.

**Figure 10:**
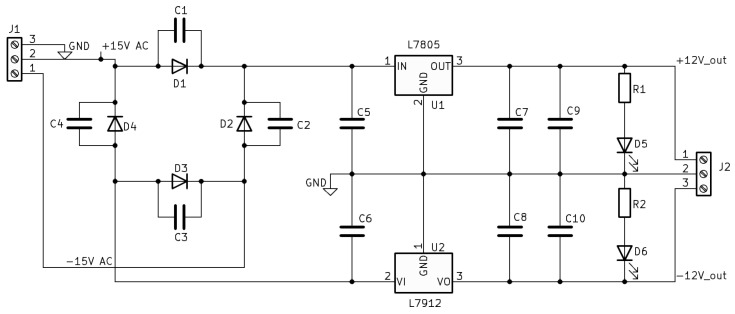
Schematic of the custom AC/DC converter for laser supply.

The laser subsystem receives 12 V from an industrial supply, which is stepped down to 5 V for the microcontroller and analog circuitry. A custom AC/DC converter (Figure 10) isolates the analog laser control from digital noise, transforming 15 V AC to ±12 V DC via a full-wave bridge and linear regulators, with LEDs for status indication.

The motion system consists of Xeryon XLS-1-40-78 motors (Xeryon AG, Zürich, Switzerland) for the X/Y axes and an XLS-3-40-78 (Xeryon AG, Zürich, Switzerland) for Z, mounted on custom aluminum parts. Motors are controlled by XD-OEM controllers (XD-OEM, AG, Zürich, Switzerland) linked to an EtherCAT motherboard, with USB control via Python (version 3.14.2, Python Software Foundation, Wilmington, DE, USA) code for simplicity. Motion performance depends on careful tuning of mass, proportional gain, frequency, speed, and tolerance parameters for each axis. Heavy loads and gravity compensation in the Z-axis require higher proportional gain and lower motor excitation frequency, while the X/Y axes are lighter and faster.

The exposure dose in direct laser writing is determined by laser power and scanning speed. Laser power was measured at the fiber output with a Thorlabs PM101 meter (Thorlabs, Inc., Newton, NJ, USA). The beam area at the focal point is $A=\pi {\left(D/2\right)}^{2}$, and the exposure dose is estimated as:

$$\mathrm{Dose}=\frac{\mathrm{Laser}\;\mathrm{Power}}{\mathrm{Laser}\;\mathrm{Area}}\times \frac{\mathrm{Laser}\;\mathrm{Diameter}}{\mathrm{Laser}\;\mathrm{Speed}}\times 100$$

where the constant factor converts units to mJ/cm^2^. For the photoresist used, the nominal dose is approximately 600 mJ/cm^2^.

Also, Table 1 shows that slower motor speeds result in an increase of the effective exposure when comparing all speeds at the same laser current. To compensate for the motor’s settling time, the speed should be as low as possible; however, lower speeds also increase the exposure dose. Therefore, a balance must be found between minimizing motor speed and keeping the laser current above the instability threshold (19 mA) to maintain a controlled exposure dose.

**Figure 11:**
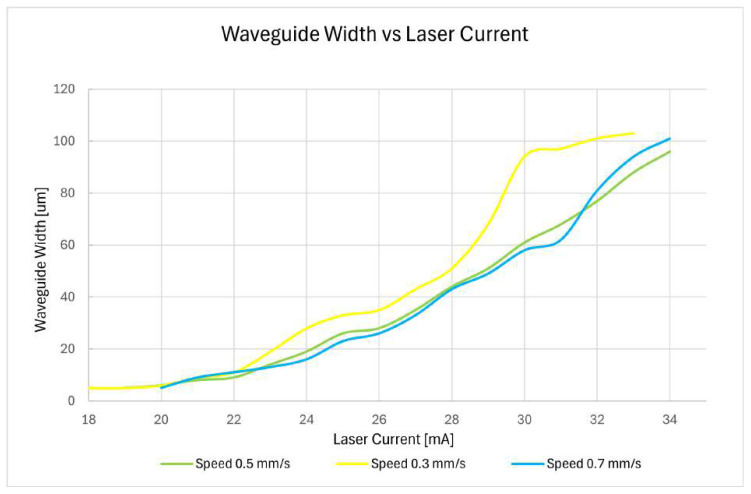
Measured waveguide width as a function of laser current for three different writing speeds: 0.3, 0.5, and 0.7 mm/s.

Figure 11 shows the experimentally measured waveguide width as a function of laser current for three different writing speeds: 0.3, 0.5, and 0.7 mm/s. The measurements were performed on a 5 μm-thick layer of SU-8 photoresist. The waveguide width depends strongly on the laser dose delivered to the photoresist, which is determined primarily by the laser current and the writing speed. Higher laser currents increase the energy delivered per unit area, while slower writing speeds allow more energy to accumulate at each point. Consequently, slower writing speeds generally produce wider waveguides for the same laser current, whereas faster speeds yield narrower waveguides. The intermediate speed of 0.5 mm/s produces waveguide widths between the other two, following the expected trend. These results indicate that the waveguide width can be effectively tuned by adjusting both the laser current and the writing speed, with the best experimental conditions yielding waveguides around 5 μm in width. This emphasizes the importance of controlling both parameters to achieve consistent and reproducible waveguide dimensions in SU-8.

### 2.4. Programming

The embedded software for the custom printed circuit board provides digital control of the laser diode, bridging the analog Thorlabs IP250-BV driver (Thorlabs, Inc., Newton, NJ, USA) with the computer application. This firmware enables precise, software-driven modulation of laser power (0–250 mA via 0–250 mV signals) and integration with safety features. Running on an STM32F103C8T6 microcontroller (STMicroelectronics, Geneva, Switzerland, Blue Pill board), it handles analog-to-digital converter multiplexing, inter-integrated circuit communication with the MCP4725 digital-to-analog converter (Microchip Technology Inc., Chandler, AZ, USA), liquid crystal display updates, and universal serial bus command processing. The firmware is implemented in C using STM32CubeIDE (STMicroelectronics, Geneva, Switzerland) and is available at https://github.com/andrerrmoreira-hue/ASERMETA (accessed on 23 December 2025).

The Blue Pill board hosts the STM32F103 microcontroller (STMicroelectronics, Geneva, Switzerland) with a USB port for virtual COM port communication. Commands are executed via a state machine that prioritizes safety. The firmware interfaces with the MCP4725 DAC (Microchip Technology Inc., Chandler, AZ, USA) to control the laser current through the IP250-BV analog board. Specific microcontroller pins are assigned as follows: PA0 monitors alarm signals, PA1 controls the laser enable input, PA2 and PA3 read temperature and current feedback, and several pins (PA6, PA7, PB0, PB1, PB10, PB11) drive a 16 × 2 LCD for real-time monitoring. Communication with the DAC and other peripherals is handled via I2C (PB6, PB7), while PA11 and PA12 manage USB communication. An analog-to-digital converter multiplexes the temperature and current signals with 12-bit resolution and a 4.5 μs sampling time.

**Figure 12:**
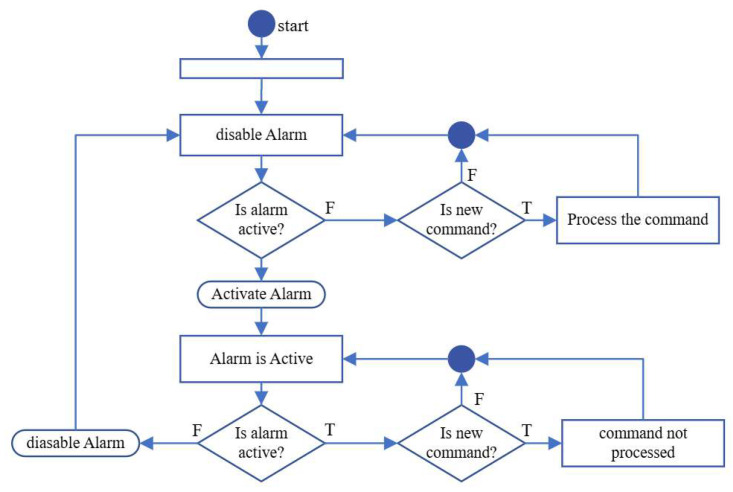
State machine of the laser digital control system.

The firmware implements a state machine (Figure 12) to manage laser operation, prioritizing safety while coordinating software commands. This architecture is necessary because the IP250-BV driver (Thorlabs, Inc., Newton, NJ, USA) is analog and lacks inherent digital interlocks. The state machine continuously monitors the PA0 pin for alarm signals, which indicate unsafe conditions such as over-temperature. When an alarm is detected, PA1 is pulsed to immediately disable the laser via the IP250-BV, preventing operation under hazardous conditions. Any commands received from the computer during this state are rejected and queued for later processing, ensuring that no unsafe instructions are executed. Once the alarm clears, PA1 is pulsed again to safely re-enable the laser, allowing normal operation to resume.

During normal operation, the state machine processes commands received via USB from the computer application. These commands include turning the laser on or off, enabling or disabling the laser driver, and setting the maximum or minimum laser current. The firmware ensures sequential execution of commands: only one command is processed at a time, while additional commands are stored in a circular buffer to prevent loss or overflow. Commands such as “turn on laser” or “turn off laser” adjust the external DAC voltage, modulating the laser current through the IP250-BV for patterning or standby operation, whereas “enable” and “disable” commands directly toggle the laser driver for full hardware-level control. By combining alarm monitoring with command sequencing, the state machine guarantees both safety and precise software-driven control, preventing the laser from operating under unsafe conditions while maintaining flexibility for real-time lithography processes.

The slicer software converts Drawing Exchange Format (DXF) design files into a sequence of executable commands used to drive the motion stages and laser during fabrication of polymer-based photonic-integrated circuits. Implemented in Python, the slicer optimizes the generated motion path according to the laser beam characteristics and mechanical constraints of the platform. The user configures the slicer through parameters such as fileStep, tolerance, beamDiameter, densifyStep, sobrePosition, and finalResolution, along with the input DXF file and desired output filename.

The slicer workflow consists of nine processing stages. First, the DXF file is parsed, and all geometric entities are extracted and converted into polygonal representations. Duplicate polygons are then removed, followed by a merging stage that joins adjacent polygons with matching dimensions to avoid unnecessary discontinuities. Next, the polygons are grouped based on spatial connectivity to reduce long travel distances during fabrication. The grouped polygons are reordered to establish an efficient writing sequence and consistent starting orientation. After reordering, polygon edges are extended when needed to ensure geometric continuity and compensate for the beam footprint. Finally, offsets are applied according to the laser beam diameter and overlap settings, generating the final exposure trajectory.

**Figure 13:**
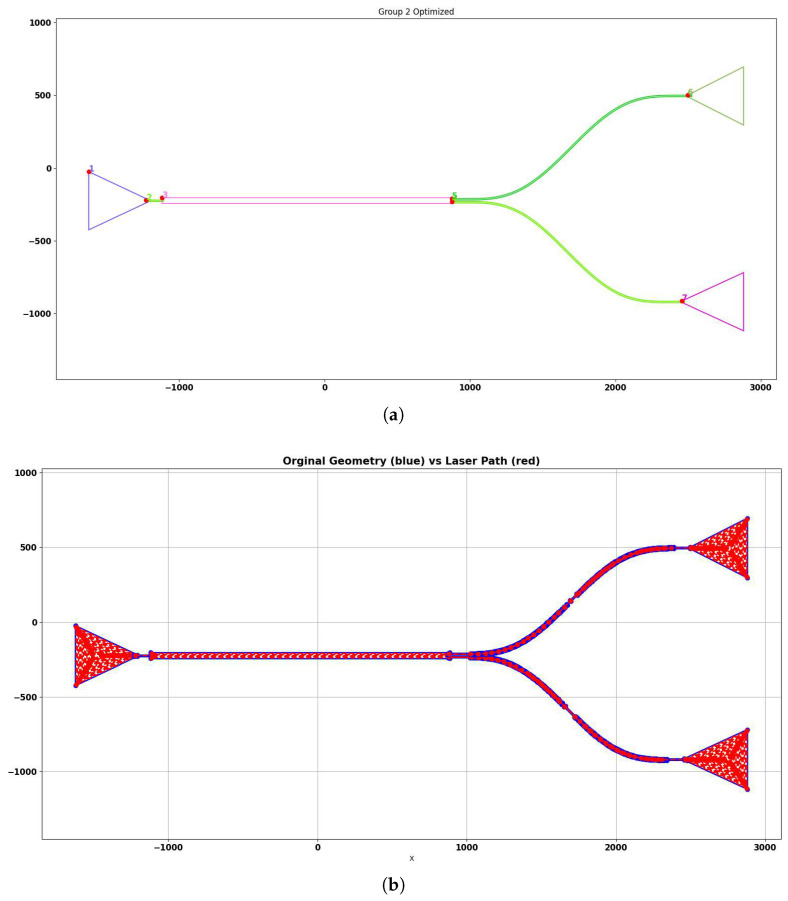
Two stages of the slicer processing pipeline: (**a**) intermediate grouped and merged polygons. Intermediate result of the slicer processing pipeline, showing the grouped and merged polygons generated from the imported DXF geometry. Different colors represent distinct writing groups optimized to minimize stage travel during fabrication. (**b**) final offset path followed by the motion stages. Final laser-writing trajectory generated by the slicer. The red line represents the complete exposure path that the motion stages will follow during fabrication, including all offset passes required to cover the full polygon width.

Figure 13a illustrates an example geometry imported from a DXF file and processed by the slicer up to the grouping stage. At this point, the original vector design has been converted into uniform polygons, merged where applicable, and organized into writing groups while preserving the functional layout of the circuit.

After grouping, the slicer computes the final laser-writing trajectory. If the polygon width is similar to the beam diameter, a single centerline path is generated by shrinking the polygon geometry. For wider features, a multi-pass exposure is created by repeatedly offsetting the polygon interior according to the configured overlap percentage. These operations are performed using custom slicing routines implemented in C for efficiency, including point densification and reduction to maintain a consistent spacing between final coordinates. The output consists of a continuous trajectory where each point includes stage coordinates, motion speed, and corresponding LaserON/LaserOFF states.

Figure 13b shows the resulting fabricated scan path in red, representing the final pattern that will be executed by the motion control system.

The graphical user interface (GUI) was developed in Python 3.12 using CustomTkinter (accessed on 23 December 2025), a modern open-source library designed for building desktop applications with clear layout control and contemporary visual styling. Unlike traditional GUI libraries that produce basic or outdated appearances, CustomTkinter provides scalable widgets, responsive window behaviour, and native light/dark theme support, allowing the interface to remain consistent across operating systems and rapid development of interactive elements such as buttons, sliders, text windows, and status indicators.

The application runs in a 1600 × 750 dark-themed window and is structured into four main panels: Status, Motor Control, Laser Control, and Program. The Status panel displays live feedback from the motors and laser, including coordinate updates and operational states. The Motor Control panel enables manual positioning and calibration with precise movement commands. The Laser Control panel provides adjustment of diode current and on/off state. The Program panel manages workflow tasks including geometry creation, DXF slicing, preview of the computed trajectory, and final execution.

**Figure 14:**
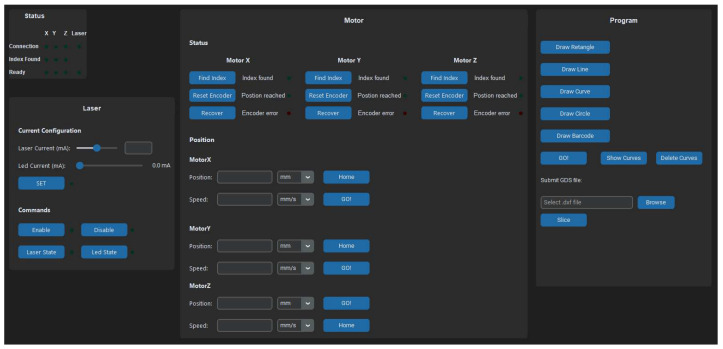
Graphical User Interface for the proposed Direct Laser Writing System.

During execution, the system automatically moves the motors to the starting position, activates the laser along the computed trajectory, and disables it upon reaching the final point, ensuring consistent and safe fabrication behavior (Figure 14).

After presenting the system interface, the effective writing speed of the DLW setup emerges as an important structural parameter, reflecting not only the nominal speed set in software but also delays introduced by the hardware. Table 2 summarizes a comparison between the theoretically expected writing time and the actual time measured for a 13 mm curved waveguide composed of 2000 points. As observed, the system consistently requires more time than predicted by the theoretical calculation. To better approximate the expected exposure and avoid overexposure, which could increase the waveguide width, the effective writing speed must be reduced.

This discrepancy is not caused by the control software, which is optimized and multithreaded to coordinate the laser and motion stages simultaneously. Instead, it arises from the settling time of the motors, which becomes significant when writing high-resolution patterns. The motor settling time limits the system’s ability to precisely follow the theoretical trajectory at high speeds, highlighting a key structural parameter: the dynamic response of the motion stages. Addressing this effect will require future work to investigate more deeply how motor parameters, such as acceleration, inertia, and PID tuning, influence the actual writing time and the resulting fidelity of the fabricated waveguides.

**Figure 15:**
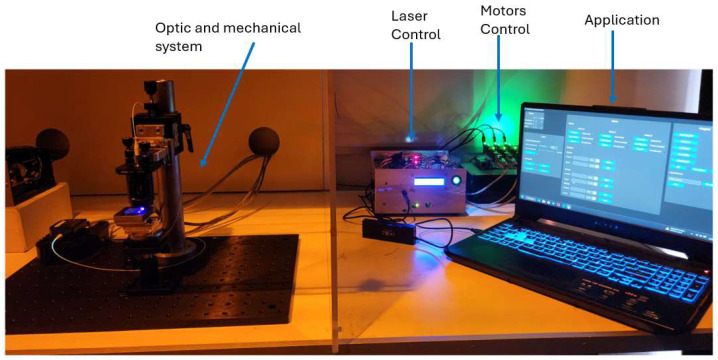
Fully assembled physical DLW system.

Figure 15 presents the fully assembled DLW system in operation for photonic-integrated circuit fabrication. The mechanical and optical components are visible, along with the hardware controlling the laser and motors. The system interfaces with the software application, which sends commands to the motors and laser, enabling overall control, monitoring of system states, and submission of fabrication files.

## 3. Results

The photonic structures presented in this study were fabricated using a custom-developed direct laser writing (DLW) system. Glass substrates were used, with a 5 μm layer of mr-DWL-5 negative photoresist. The glass side was chosen to minimize the refractive index mismatch with the photoresist, allowing for larger, easily observable features suitable for initial prototyping. Spin coating and soft baking parameters were optimized to achieve uniform layers across the substrate.

Patterns were written using the DLW system, with laser power and scan speed adjusted to achieve consistent feature sizes. Although the system currently lacks a feedback mechanism to ensure precise focus, careful calibration allowed reproducible patterning across multiple substrates. The custom setup enabled basic edge-coupling measurements for functional verification of fabricated waveguides.

To evaluate the relationship between laser dose and feature dimensions, a series of waveguides were written on a flat glass substrate with 5 μm-thick photoresist. The scan speed was held constant at 0.3 mm/s, while the laser current was varied. Optical power was measured at the fiber output, and the corresponding exposure doses were calculated Table 3.

Below 18 mA, polymerization was incomplete, while higher currents produced progressively wider features. The resulting data allowed calibration of laser parameters to achieve consistent, reproducible line widths. Although the effective doses were higher than manufacturer specifications due to non-ideal focus, the system reliably produced features suitable for functional photonic circuits.

**Figure 16:**
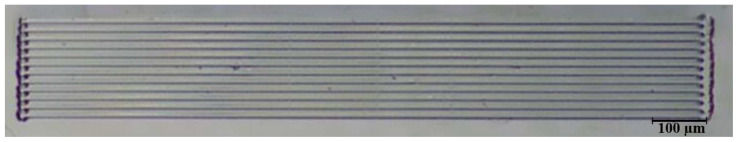
Diffraction grid fabricated with the proposed DLW system.

The DLW system was validated by fabricating several photonic structures. First, a diffraction grid consisting of 10 μm lines with 10 μm spacing was produced, demonstrating the system’s patterning capabilities (Figure 16).

**Figure 17:**
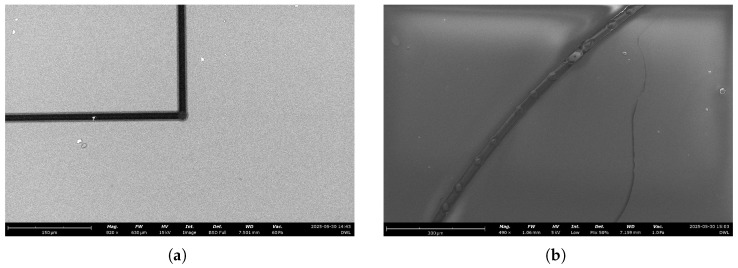
SEM images of waveguides fabricated with the DLW system. (**a**) 90° corner; (**b**) Curved guide.

SEM analysis of a curved waveguide showed a thickness of approximately 5 μm and slightly rounded corners due to the Gaussian profile of the laser (Figure 17a). Features were reproducible, confirming effective control over stage synchronization and exposure parameters.

A curved guide written under similar conditions further demonstrated accurate stage motion and laser control (Figure 17b). Minor intensity variations along the curve were attributed to stationary periods of the motors during stage repositioning, highlighting areas for future system optimization.

An MMI (multimode interference device) device was fabricated to validate the DLW system’s capability for producing functional photonic circuits. It operates on the principle of self-imaging: light entering a wide multimode waveguide excites multiple modes that interfere constructively and destructively, reproducing the input field at specific distances along the device. This allows for compact and low-loss power splitting, making MMIs widely used in applications such as optical communications and sensing. The MMI used in this study was designed as a 1 × 2 splitter for visible red light (605 nm) with a 50:50 power division. Its central section measures 40 µm in width and 2000 µm in length, with input and output waveguides and a photoresist thickness of 5 µm.

**Figure 18:**
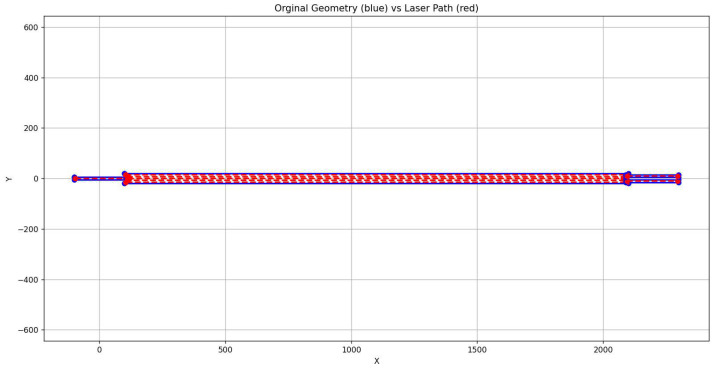
Laser-writing trajectory generated by the slicer for the MMI device. The red lines represent the full set of paths where the laser is activated, covering both the input/output waveguides and the central 40-µm-wide, 2000-µm-long MMI region.

The design, prepared externally, was exported as a DXF file and submitted to the slicer software. The slicer transformed the fabrication file into executable commands for the motors and laser. The resulting laser trajectory for the MMI, showing the complete pattern that the DLW system would follow during fabrication, is presented in Figure 18. The red lines indicate the paths where the laser will be activated, demonstrating smooth coverage of both the waveguides and the central MMI region.

**Figure 19:**
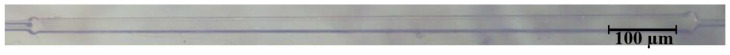
Fabricated MMI using the DLW system.

The fabricated device was then produced using the DLW system, following the optimized laser paths generated by the slicer. The resulting structure, shown in Figure 19, exhibits clearly defined waveguides and a well-formed central MMI region. The precise edges and uniform exposure confirm that the system can accurately reproduce complex geometries from externally prepared designs, validating its readiness for polymer-based photonic-integrated circuit fabrication.

**Figure 20:**
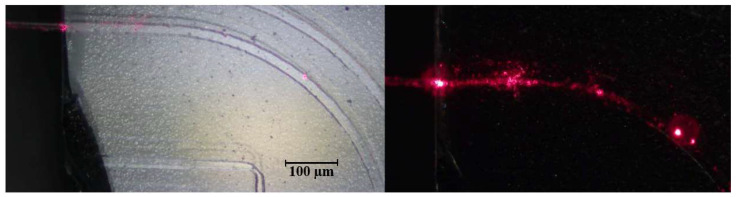
Insertion of light in a curve fabricated with the proposed DLW system.

The fourth and final test involved fabricating a simple curve structure using the basic geometries tab in the GUI, with a width of 15 μm and a thickness of 5 μm, matching the photoresist layer. Light was successfully inserted via edge coupling, demonstrating that the complete workflow—including substrate preparation, fabrication, and analysis—operates correctly. The faint scattering observed along the curve reflects minor imperfections, but the experiment confirms that the DLW system can produce functional photonic structures and supports practical evaluation of light propagation (Figure 20).

For PIC fabrication, only qualitative light transmission observations are presented. These observations confirm that the fabricated waveguides are functional and guide light as expected. Quantitative optical measurements, such as transmission loss, were not performed, as the primary focus of this work is on the development and demonstration of the ASERMETA direct laser writing system. The results emphasize the system’s capability to fabricate waveguides with high geometric flexibility and reliable feature sizes, while detailed optical characterization will be addressed in future studies focused on device performance.

Overall, these results demonstrate that the DLW system can reliably fabricate complex photonic structures in a standard academic laboratory environment. While precise focal control and environmental stability remain challenges, the system enables rapid prototyping and functional testing of polymer-based photonic-integrated circuits. Notably, no anti-reflective coating was applied to the substrate during these experiments; although such coatings could improve performance, this choice was made to isolate and evaluate the system’s capabilities based solely on the hardware performance.

## 4. Conclusions

This work presented the development of an affordable direct laser writing (DLW) system tailored for academic laboratories, enabling the fabrication of polymer-based photonic-integrated circuits (PICs) without requiring costly infrastructure. The system integrates mechanical, optical, electronics, and software subsystems into a functional and compact platform capable of producing reproducible photonic structures.

The DLW system was validated through the fabrication of diffraction grids, curved waveguides, and a multimode interference (MMI) device, demonstrating its ability to accurately pattern functional photonic circuits. Despite limitations in focal control, environmental stability, and substrate flatness, the system achieved a resolution of approximately 10 μm, highlighting its potential for rapid prototyping in research environments. Custom-developed software, including a hybrid slicer and Python-based GUI, enabled flexible design import, parameter adjustments, and execution of complex patterns, making the system accessible to researchers with varying levels of experience.

Overall, this project represents not only the creation of a DLW system but also the establishment of the associated fabrication and analysis processes, resulting in ISEL’s first operational polymer-based PIC. With further improvements, such as environmental control and automated focal adjustment, the system could achieve higher resolution and enhanced reproducibility, paving the way for broader adoption of affordable photonic prototyping in academic settings.

## Figures and Tables

**Table 1:** Measured laser power and calculated exposure dose for different laser currents and scanning speeds.

Current [mA]	Power [mW]	Dose [mJ/cm^2^]		
		Speed = 0.7 mm/s	Speed = 0.5 mm/s	Speed = 0.3 mm/s
16	0.002	364	509	849
17	0.003	546	764	1273
18	0.005	909	1273	2122
19	0.006	1091	1528	2546
20	0.010	1819	2546	4244
21	0.052	9458	13,242	22,069
22	0.196	35,651	49,911	83,185
23	0.368	66,936	93,710	156,184

**Table 2:** Expected vs. real times for different writing speeds.

Speed [mm/s]	Expected Time [s]	Real Time [s]	Time Difference [s]
0.3	45.53	70.63	25.10
0.4	34.15	66.22	32.07
0.5	27.32	57.00	29.68
0.6	22.77	50.92	28.15
0.7	19.51	46.97	27.46
0.8	17.08	44.21	27.14
0.9	15.18	43.40	28.22
1.0	13.66	42.53	28.87
2.0	6.83	39.52	32.69
3.0	4.55	39.43	34.88

**Table 3:** Relation between laser current, optical power, calculated dose, and resulting line thickness at a fixed speed of 0.3 mm/s.

Current (mA)	Power (mW)	Line Width (µm)	Dose (mJ/cm^2^)
17	0.003	–	1273
18	0.005	5	2122
19	0.006	5	2546
20	0.010	6	4244
21	0.052	9	22,069
22	0.196	11	83,185
23	0.368	19	156,184
24	0.561	28	238,096
25	0.744	33	315,763
26	0.930	35	394,704
27	1.129	43	479,162
28	1.310	51	555,981
29	1.497	68	635,347
30	1.676	94	711,316
31	1.854	97	786,862
32	2.049	101	869,623
33	2.238	103	949,837
34	2.437	107	1,034,295

## Data Availability

The data supporting the findings of this study are openly available in the repository https://github.com/andrerrmoreira-hue/ASERMETA (accessed on 23 December 2025).
